# Unexpected Effects of Sulfate and Sodium Chloride Application on Yield Qualitative Characteristics and Symmetry Indicators of Hard and Soft Wheat Kernels

**DOI:** 10.3390/plants12050980

**Published:** 2023-02-21

**Authors:** Tatiana S. Aniskina, Ekaterina N. Baranova, Svyatoslav V. Lebedev, Nelli S. Reger, Ishen N. Besaliev, Alexander A. Panfilov, Viktoriya A. Kryuchkova, Alexander A. Gulevich

**Affiliations:** 1 N.V. Tsitsin Main Botanical Garden of Russian Academy of Sciences, Botanicheskaya 4, 127276 Moscow, Russia; 2 All-Russia Research Institute of Agricultural Biotechnology, Timiryazevskaya 42, 127550 Moscow, Russia; 3 Federal Scientific Center of Biological Systems and Agrotechnology of the Russian Academy of Sciences, 9 Yanvarya 29, 460000 Orenburg, Russia

**Keywords:** wheat kernel, salt effect, NaCl, Na_2_SO_4_, kernel phenotype, fluctuating asymmetry

## Abstract

The heterogeneity of grain quality can lead to limited predictability of qualitative and quantitative characteristics of the wheat yield, especially with an increase in the importance of drought and salinity caused by climate change. This study was undertaken with the aim of creating basic tools for phenotyping and assessing the sensitivity of genotypes to salt effects at the level of some wheat kernel attributes. The study considers 36 variants of the experiment, including four wheat cultivars—Zolotaya, Ulyanovskaya 105, Orenburgskaya 10, Orenburgskaya 23; three treatment variants—control (without salt) and two salts exposure (NaCl at a concentration of 1.1 g L^−1^ and Na_2_SO_4_ at a concentration of 0.4 g L^−1^); as well as three options for the arrangement of kernels in a simple spikelet—left, middle, and right. It has been established that the salt exposure had a positive effect on the percentage of kernel fulfilling in the cultivars Zolotaya, Ulyanovskaya 105, and Orenburgskaya 23 compared to control. The kernels of the Orenburgskaya 10 variety matured better in the experiment with Na_2_SO_4_ exposure, while the control variant and NaCl gave the same effect. When exposed to NaCl, significantly greater values of weight, transverse section area, and transverse section perimeter of the kernel were noted in the cv Zolotaya and Ulyanovskaya 105. Cv Orenburgskaya 10 responded positively to the use of Na_2_SO_4_. This salt caused an increase in the area, length, and width of the kernel. The fluctuating asymmetry of the left, middle, and right kernels in the spikelet was calculated. In the cv Orenburgskaya 23 the salts affected only the kernel perimeter among parameters examined. The indicators of the general (fluctuating) asymmetry were lower in the experiments with the use of salts, i.e., kernels were more symmetrical than in the control variant, both for the cultivar as a whole and when compared taking into account the kernel location in spikelet. However, this result was unexpected, since salt stress inhibited a number of morphological parameters: the number and average length of embryonic, adventitious, and nodal roots, flag leaf area, plant height, dry biomass accumulation, and plant productivity indicators. The study showed that low concentrations of salts can positively affect the fulfilling of kernels (the absence of a cavity inside the kernel) and the symmetry of the left and right sides of the kernel.

## 1. Introduction

Wheat is the most important crop in the world; it provides the main food in most regions of the planet and is sown on more than 220 million hectares [1]. A variety of genotypes and growing conditions allows breeders to create varieties with different types of resistance, different features of ontogenesis, and, hence, different qualitative characteristics of the most valuable wheat product—single-seeded fruits or caryopses (kernels) [2]. The formation of a kernel is a complex process that includes many transformations of various tissues in the developing wheat fruit. These processes occur over several weeks and include both the death of a number of tissues and the formation of new tissues that form the kernel (caryopsis) and its integuments [3,4]. The impact of abiotic factors on such a system should hypothetically cause significant changes both in the formation processes themselves and in the storage processes in specialized tissues such as the endosperm and the aleurone layer.

The structure of the wheat spike creates a deceptive feeling of the presence of bilateral symmetry [5]. The features of the spiral geometry characteristic of the reproductive organs, as well as for the entire wheat plant as a whole, become apparent only with a detailed study of ontogenesis and the sequential transformation of the phenotypic pattern of the flowering organ from the primary tubercle to a full-fledged multi-spikelet inflorescence, traditionally described as a spike (ear) [6]. The very sequence of laying flowers in spikelets makes it obvious that there is a certain underlying asymmetry in this seemingly ideal model—the kernel. The performance of asymmetry in the spikelets and spikes of several wheat genotypes with different characteristic arrangement of kernels in the ear was described earlier [7]. The physical reasons for the asymmetry of kernels were predicted at the beginning of the 20th century [8] and have not yet been experimentally confirmed due to the complexity of the qualitative setting of the experiment and the lack of an explicit experimental approach [9]. Meanwhile, a higher degree of symmetry is considered by breeders as an indication of a genetic advantage [10].

Climate change in the near future will lead to a significant reduction in yields due to increased adverse impacts such as drought, excessive humidity, and primary and secondary soil salinization [11]. Currently, salinity is of concern due to the fact that sodium salts are not able to form stable insoluble compounds and often cause an increase in osmotic potential and accumulation of ions to critical concentrations that damage plant roots when accumulated by groundwater during reclamation and irrigation [12]. Additionally, in some cases, salinization can occur due to the use of road de-icing agents, which are then washed out into the environment [13,14]. In regions close to large saline lakes, seas, or oceans, this effect can be caused by climate change causing salt transport by wind [15], droughts [16], and storms from the sea leading to salinization of coastal wetlands [17,18].

Wheats with a different genotype and origin may show different tolerance and sensitivity to salinity [19,20,21]. The effects of the action of salts on seedlings, on plant biomass, on the ability for effective photosynthesis are exceedingly well studied; changes are shown at the ultrastructure level of the nucleus, plastids, and cytoskeleton [22,23]. Salinity causes intracellular processes characteristic of oxidative, osmotic, and toxic stress. Cells cannot cope with the excess supply of toxic sodium ions; as a result, intra- and intercellular transport in various parts of the plant is disrupted. A specific system of various transporters causes a redistribution of other ions, disrupting the processes of synthesis, transport, and accumulation of primary metabolites and stored compounds. Additionally, significant damage is observed in the activity of a number of important enzymes and gene expression [19,24]. However, the final results for evaluating the productivity, namely, the quality characteristics of the grains and the yield, are important. Selection and forecasting require both qualitative and quantitative evaluation methods capable of predicting the consequences of using a variety or breeding line. It is possible that the assessment of the phenotype of kernels in the spike can become reliable evaluation criterion in the future agronomic studies of wheat cultivated in saline areas.

The present study was carried out to apply the previously estimated indicators of quantitative determination of the degree of kernel asymmetry in two cultivars of soft wheat and two cultivars of durum wheat when cultivated in the field under salinity conditions due to sulfate and sodium chloride. In addition, the goal was to evaluate the quality indicators and the fluctuating asymmetry in the kernels.

## 2. Results

### 2.1. Salt Exposure Effect on Morphological Parameters of Wheat Plants

Due to the presence of moisture in the root layer and precipitation that fell in the initial period of development (shooting–tillering) of spring wheat, the root systems of the studied cultivars were well developed. They consisted of both primary (embryonic and adventitious) and secondary (nodal) roots. The embryonic roots were subject to a more significant decrease under the influence of salinity, with the greatest manifestation in the cv Orenburgskaya 23 and Zolotaya (Table 1). The cv Orenburgskaya 10 was more stable in this indicator. In the first two cultivars, not only their number decreased, but also the average length. In the cv Ulyanovskaya 105, only the number of embryonic roots decreased.

Under the influence of various salinity options, the number of adventitious roots decreased in cultivars Ulyanovskaya 105, Orenburgskaya 10, and Zolotaya. Salt exposure caused a decrease in the number of nodal roots in the cv Ulyanovskaya 105.

Differences in the suppressive effect of chloride and sulfate salinization are insignificant and are not clearly marked, taking into account the least significant difference (LSD) for the 5% significance level.

A decrease in plant height as a factor in the inhibitory effect of salt stress was manifested in three cultivars (Table 2). In cultivars Orenburgskaya 23 and Ulyanovskaya 105, it was noted under the influence of Na_2_SO_4_ treatment. In the cv Orenburgskaya 10, both types of salinity contributed to the decrease in plant height. The decrease in the length of the spike in cultivars Orenburgskaya 23, Ulyanovskaya 105, and Orenburgskaya 10 was largely due to the negative effect of chloride salinity. For the cv Zolotaya, no negative effects of additional salinization on the length of the spike were noted, and even a positive effect of chloride salinization on this indicator was noted.

### 2.2. Effect of Salts on Fulfilling (Plumpness) of Kernels

The study revealed that out of 251 kernels examined in cv Zolotaya plants, only 50 kernels were incompletely matured or had damage. In the control variant, the percentage of unmatured kernels was 33% from the sampling (38 out of 114 kernels), while in the variants with the treatment by salts, the result differed in a positive direction—10% with Na_2_SO_4_ and 7% with NaCl. When comparing the percentage of matured, well-filled kernels among the left and right kernels in spikelet, it turned out that the left kernels have a greater percentage in all salinity options: 73% in the control (61% of the maturing in the right kernels), 94% when exposed to NaCl (89% of the right kernels), and 92% under exposure to Na_2_SO_4_ (88% of the right kernels).

A similar result in maturation was noted in the cv Ulyanovskaya 105. Thus, in the variant with application of NaCl, 98% of the kernels (130 out of 133 kernels) were matured, under Na_2_SO_4_ exposure 91% of the kernels (150 out of 164 kernels) were matured, and in the control variant 72%. In total, 466 kernels were examined in the study, and 401 of them (86%) were maturing (well-filled). Upon a detailed examination of the left and right grains, it should be noted that in the experiment with NaCl exposure, 100% of the left grains and 95% of the right kernels were maturing, while under exposure to Na_2_SO_4_—93% of the right kernels and 88% of the left kernels, and in the control 73% of the left kernels and only 65% of the right kernels were maturing.

Cultivar Orenburgskaya 23 matured better in the variant with exposure to NaCl (92% of matured or 96 of 104 kernels), in the experiment with exposure to Na_2_SO_4_—88% (78 of 89 kernels), and in the control variant 83% (95 of 115 kernels). In total, in the sample of 308 kernels examined, 269 matured, well-filled kernels (87%) were noted. The proportion of the distribution of matured grains relative to their location in the spikelet after exposure to salts on wheat plants was as follows: in the variant with NaCl, 93% of the right and 93% of the left kernels were maturing; in the variant with Na_2_SO_4_,—90% of the right and 83% of the left kernels; and in the control variant without salt impact—77% right and 84% left kernels were maturing.

Approximately the same result in maturation of kernels is observed in the cv Orenburgskaya 10 in the control variant and the variant with the use of NaCl—69% and 68%, respectively. However, the best result was obtained in the experiment with Na_2_SO_4_, where fully matured kernels accounted for 78%. In general, the sampling consisted of 147 kernels, of which 42 were damaged or unmatured. Salt exposure caused greater maturation of the right kernels in spikelets (71% of the right and 57% of the left ones in the variant with NaCl, and 86% of the right and 69% of the left ones in the variant with Na_2_SO_4_), while in the control variant more left kernels in spikelets were matured (72% of the left and only 58 % right ones).

### 2.3. Effect of Salts on the Main Parameters of Kernels

**Figure 1:**
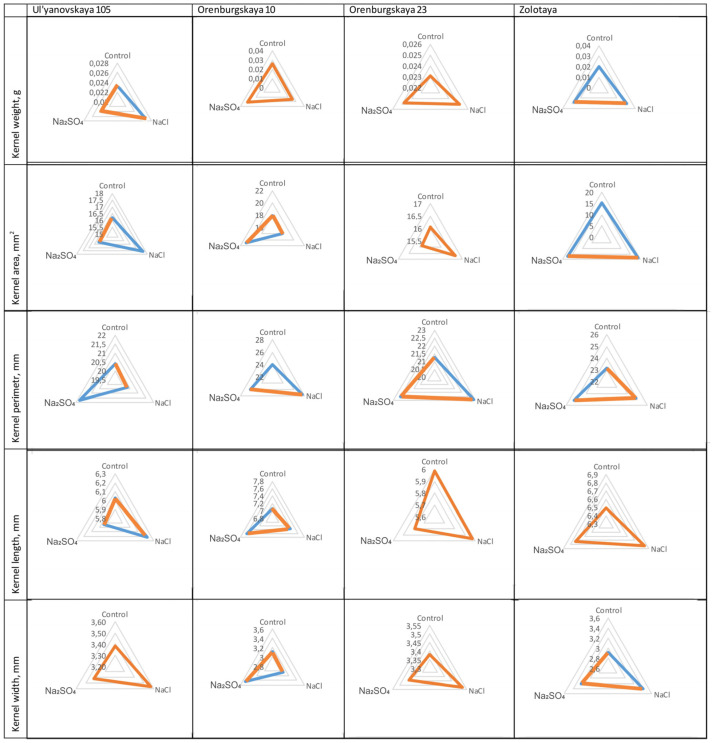
The examined kernel parameters in various wheat cultivars exposed to salt treatment in field conditions. The results are represented as radar charts. Significant differences are indicated in blue, non-significant differences in orange.

The Kruskal–Wallis test confirmed the hypothesis of the effect of salts on the weight, area, perimeter, and width of the kernel, except for the length of the kernels in the cv Zolotaya (Table 3). The weight of kernels in the cv Zolotaya plants exposed to NaCl (average kernel weight 0.0305 ± 0.0123 g) and Na_2_SO_4_ (0.0281 ± 0.0143 g) does not have significant differences; however, it is significantly higher than in the control variant (0. 0199 ± 0.0141 g). The differences with the control in terms of kernel area are similar: the average area in the control variant was 15.22 ± 0.54 mm^2^, while when exposed to NaCl it was 18.73 ± 0.76 mm^2^, and to Na_2_SO_4_ was 17.41 ± 0.59 mm^2^ (Figure 1). According to the “perimeter” parameter, there are significant differences in kernels only between the control (23.16 ± 0.53 mm) and plants exposed to Na_2_SO_4_ (25.20 ± 0.55 mm), in contrast to NaCl (24.84 ± 0.57 mm), and in the kernel width—only between the control (2.92 ± 0.07 mm) and NaCl (3.41 ± 0.09 mm)-exposed plants.

Exposure to salts did not have a significant effect on the weight of kernels of the cv Orenburgskaya 10 (control—0.0262 ± 0.0024 g, NaCl—0.0254 ± 0.0017 g, Na_2_SO_4_—0.0311 ± 0.0024 g); however, an effect on other parameters was noted. Significant differences were observed both in the area of kernels due to the effect of salts (NaCl—17.94 ± 0.67 mm^2^, Na_2_SO_4_—20.97 ± 0.89 mm^2^), and in width (NaCl—3.07 ± 0.89, Na_2_SO_4_—3.47 ± 0.12). The values of the kernel perimeter in the control variant significantly differ from the results with salt exposure (control—23.97 ± 0.69 mm, NaCl—27.60 ± 0.65 mm, Na_2_SO_4_—26.12 ± 0.55 mm). A shorter kernel length was noted in the control variant—7.07 ± 0.16 mm, and a longer length in the experiment with Na_2_SO_4_—7.61 ± 0.13 mm; there were significant differences between these variants.

The differences between the control and NaCl in kernel weight (control 0.0232 ± 0.0008 g, NaCl 0.0266 ± 0.0008 g) were noted in the cv Ulyanovskaya 105. According to the parameter “kernel area”, the values of the control variant and exposure to Na_2_SO_4_ were similar (control—16.21 ± 0.35 mm^2^, Na_2_SO_4_—16.15 ± 0.28 mm^2^), but both values were less than in the variant with NaCl (17.60 ± 0.32 mm^2^). There were also no differences between the control and NaCl in terms of such an indicator as the perimeter of the kernels (control—20.40 ± 0.29 mm and variant with NaCl—20.32 ± 0.31 mm), while a greater result was achieved in the experiment with Na_2_SO_4_ (21.77 ± 0.32 mm). The kernel length values differed in the experiment with Na_2_SO_4_ (5.94 ± 0.05 mm) and NaCl (6.22 ± 0.06 mm), and salinity factors did not affect the kernel width (control—3.38 ± 0.05 mm, NaCl—3.57 ± 0.04 mm, and Na_2_SO_4_—3.42 ± 0.04 mm).

The least influence of salts on the parameters of kernels was noted in the cv Orenburgskaya 23. Thus, there was no significant effect on the weight of the kernel (control—0.0231 ± 0.0010 g, NaCl—0.0251 ± 0.0009 g, Na_2_SO_4_—0.0248 ± 0.0011 g), transverse section area, length, and width of kernels. However, there was an effect on the perimeter of the kernel transverse section, where the values differed significantly between the control (21.26 ± 0.29 mm) and the variant with NaCl (22.91 ± 0.38 mm).

### 2.4. Effect of Salts on the General Asymmetry of Kernels

It has been established that the average value of the general (fluctuating) asymmetry in the cv Zolotaya was higher in the control variant; moreover, more even kernels were observed in the variants with Na_2_SO_4_ and NaCl exposure (Table 4). The Kruskal–Wallis method revealed significant differences between the control variant and Na_2_SO_4_ exposure on this trait.

### 2.5. Effect of Salts on the Asymmetry of Left, Right, and Middle Kernels

**Figure 2:**
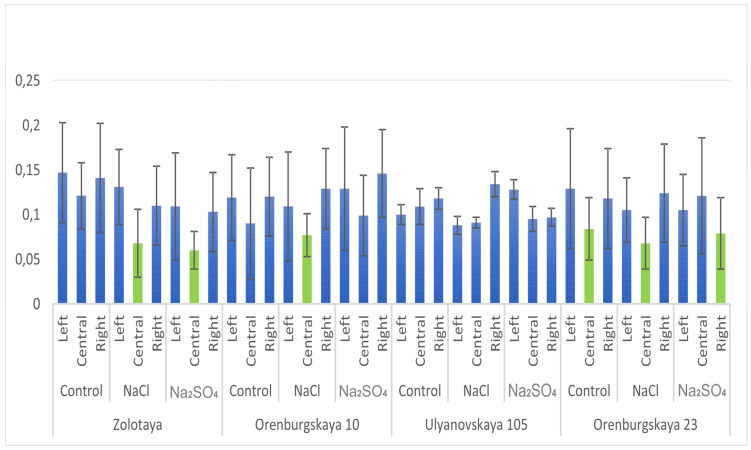
Average values of the total (fluctuating) asymmetry of kernels for wheat cultivars with different salinity variants, depending on the location in the spikelet.

In the control variant, cv Zolotaya had no significant differences (Kruskal–Wallis method) in the overall asymmetry between the left, right, and middle kernels (Figure 2). In the variant with NaCl exposure, the left kernels (0.131 ± 0.042) did not differ from the right ones; however, the left kernels significantly differed from the middle ones (0.068 ± 0.038). The result of the experiment with Na_2_SO_4_ exposure did not reveal any differences between the left kernels (0.109 ± 0.060) and the right ones (0.103 ± 0.044); however, both left and right kernels differed from the middle ones (0.060 ± 0.021). In general, kernels looked more symmetrical in the experiment with Na_2_SO_4_.

In the cv Orenburgskaya 10, there were no differences between the left, right, and middle kernels in terms of overall asymmetry in the control variant, as well as in the experiment with Na_2_SO_4_, where the asymmetry level was 0.129 ± 0.069 for the left kernel, 0.146 ± 0.049 for the right kernel, and 0.099 ± 0.045 for the middle one. In the experiment with NaCl, the middle (0.077 ± 0.024) and right (0.129 ± 0.045) kernels differed, but did not differ from the left ones.

The right, left, or middle kernels in the spikelet were evaluated (arithmetic mean ± standard deviation, *p* = 0.05). Light green color indicates variants that have significant differences with other experimental variants that were established after applying the Kruskal–Wallis method.

The Kruskal–Wallis analysis of variance did not reveal the effect of the kernel arrangement in the cv Ulyanovskaya 105 and Orenburgskaya 23 on the asymmetry level in the control variant and in the experiment with Na_2_SO_4_ exposure. Therefore, there were no differences between kernels. However, in the cv Ulyanovskaya 105, the asymmetry of the left (0.088 ± 0.010) and right (0.134 ± 0.014) kernels in the experiment with NaCl has significant differences, and in the cv Orenburgskaya 23 the asymmetry of the middle kernels (0.068 ± 0.029) significantly differs from the extreme ones. The most symmetrical kernels of the cv Ulyanovskaya 105 were noted in the right row of the spikelet in experiment with Na_2_SO_4_.

### 2.6. Scale of Fluctuating Asymmetry

Fluctuating asymmetry coefficients range from 0.016 to 0.315 in this study (Table 5). In a recent study of the fluctuating asymmetry in wheat cultivars Zlata, Agata, and Rubezhnaya, a scale for measuring the strength (degree) of asymmetry was proposed [7]. However, the upper interval ended with asymmetry values from 0.150 to 0.184, so it could not include newly obtained data. Using the initial data from the previous work (278 asymmetry coefficients for three experiment variants) and the current 461 coefficients for all experiment variants, the following measurements were proposed, which are shown in Table 5 and Table 6.

### 2.7. Determination of the Main Factors of Sampling Variability

Principal component analysis identified four main factors (components) that explain the cumulative variance of sampling. The sampling variability of 51.98 % is explained by the influence of the first component, which consists of kernel size parameters: transverse section area, transverse section perimeter, length, width, length of the symmetry axis (kernel thickness), and index 4. The second component (14.77 %) is responsible for the left-sided shape displacement (index 2 and index 5), and the third one (10.06 %) is responsible for the right-sided displacement (index 1 and index 6). The fourth component consists of index 3, which is responsible for the displacement of the grain width under the hollow triangle of the groove, and explains 9.34 % of variance of the sampling. Altogether, four components explain the variability of 86.15 % from the sampling size.

## 3. Discussion

The study of the effect of salt stress on the parameters of roots and aboveground biomass is consistent with data from other studies. As in our case, salt treatment led to a decrease in aboveground biomass and root length of wheat [25,26]. However, it was unexpected that with the suppression of the vegetative organs in comparison with the control variant, we will achieve an improvement in the studied parameters of the grain. When simulating salt stress in the studied varieties, a greater yield of the proportion of mature kernels was observed, and the values for weight, area, perimeter, width, and symmetry of wheat caryopses were also significantly higher.

**Figure 3:**
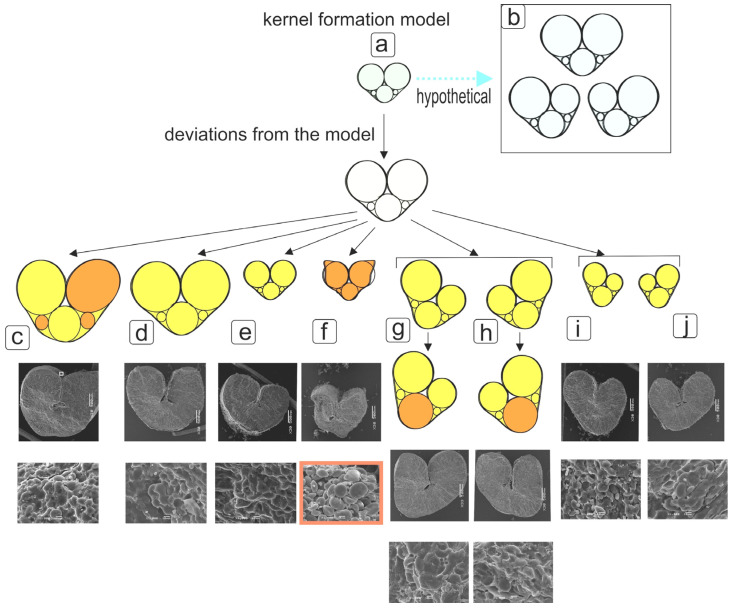
Scheme for testing the hypothesis about the stability of the dimension of conditional elements of the kernel during the formation of a kernel with manifested asymmetry, as well as hypothesis about the relationship of low-quality kernels (insufficient plumpness (fulfillment)) with the asymmetry of kernels. Designations: a—initial stage of kernel (caryopsis) formation, after the formation of the aleurone layer and endosperm; b—idealized model for the formation of symmetrical and asymmetric kernels in spikelets; c—large kernel from the lower tier with a manifested asymmetry; d—medium-sized kernel from the upper layer with a relatively symmetrical shape; e—small kernel of the upper tier with a relatively symmetrical shape; f—a small kernel from the upper tier with a manifested asymmetry and a lesion of the smoothed shape of the surface; g, h—asymmetric kernels from the lower tier of the spikelet with a manifested increase in one half and a decrease in the second one; i, j—small asymmetric kernels from the upper tier of the spikelet with a manifested increase in one half and a decrease in the second one.

There are basically two types of changes in the degree of asymmetry in response to stress—either an increase in asymmetry, or no reaction at all. In our case, low salt concentrations in 5 out of 36 variants (Figure 3) “worked uniquely”—significantly reduced the asymmetry. Previously, such an effect was observed only when studying the leaves of different cohorts of *Quercus ilex*, it turned out that plants living in more stressful places (excessive moisture conditions) are more symmetrical [27]. In rodents *Peromyscus maniculatus*, it was found that after a natural disaster (destruction of a forest reserve by a tornado), fluctuating asymmetry in the length of the femur became lower [28].

Often, external stresses led to an increase in the asymmetry of individual plant organs. Thus, the fluctuating asymmetry of birch leaves is affected by environmental pollution due to emissions from copper smelters [29], height of growth [30], excess nitrogen on *Betula pubescens* [31], interspecific hybridization on the leaves of *Betula nana*, *B. pubescens,* and *B. pendula* [32]. Leaf asymmetry is enhanced by exposure to electromagnetic fields in soybean when placed under high-voltage power lines generating pulsed magnetic fields from 3 to 50 mG [33], in *Salix borealis* due to exposure to leaf beetles *Melasoma lapponica* around the copper smelter [34], in a population of *Clarkia tembloriensis* due to inbreeding [35], in *Phaseolus vulgaris* due to water deficiency [36], in *Dimorphotheca sinuata* due to UV exposure [37], in *Lythrum salicaria* due to excess nutrients [38].

There are also works that have shown that the above dependence is not always the case. The impact of stresses (water deficit, pathogen attack, and competition) on *Salix sericea* and *Salix eriocephala* slightly changed the fluctuating leaf asymmetry. However, this study found a strong negative correlation between plant biomass and fluctuating asymmetry, i.e., plants with symmetrical leaves are better able to protect themselves from stress [39]. Mountain birch does not have asymmetry associated with insect damage [28] and pollution concentration, but is sensitive to cold conditions of the year [40]. No relationship has been found between a wide NaCl concentration gradient on *Glycine max* and fluctuating asymmetry [41], nor between asymmetry and fitness components in *Brassica cretica* [42].

Heterospermia of cereal grains is an important quality indicator that can significantly affect the properties and composition of the resulting product and products of its processing [43]. The reasons that cause the appearance of different-quality seeds (grains) can be genetic, that is, characteristic of the variety, but can be induced by cultivation features: humidity, temperature, nutrient composition, acidity, predominant air movement, and the spectral composition of light and its intensity. Additionally, a similar effect should be expected from features associated with the location: gravitropism or exposure to a magnetic field or atmospheric pressure. A special factor may be the impact of pests and diseases, in which the parameters of symmetry and asymmetry of grains (kernels) in the spike will be significantly distorted.

Meanwhile, the predictability of qualitative indicators is extremely important for creating modern foundations for predictive phenotyping and analysis of factors causing changes in the qualitative and quantitative characteristics of grain, which is a prospect for the near future [44,45]. If the heterogeneity of the upper, smaller kernels in each spikelet, and the lower, respectively, larger kernels, can be used, for example, by separating small and large kernels using sieves or using differences in their mass, then taking into account and eliminating changes in characteristics associated with symmetry and asymmetry of kernels conditionally upper and conditionally lower in each spikelet mechanically is currently not possible. Nevertheless, qualitative differences between both small and large kernels and between relatively symmetrical and asymmetric kernels are obvious. They are caused by differences in the ratio of the volume of the germ, endosperm, and aleurone layer, which allows us to predict the quality of flour for specific purposes [46]. Obviously, in small grains, the ratio of protein to starch will be higher than in large ones, since the larger the volume of the endosperm, the more starch in the grain and the smaller the number of cells of the aleurone layer, which is one layer of cells around the endosperm [47]. It is likely that the noted effects may be accompanied by a change in the biosynthesis or distribution of some plant hormones, for example, auxins [5]. With pronounced genetic or adverse effect-induced asymmetry, unevenness already occurs at the level of each kernel (grain), which can create problems not only during processing, but also during storage. Thus, a grain with cavities can have altered respiration [48], and consequently lipid and protein oxidation [49], which can affect the duration of viability during storage and subsequent germination [50]. The development of kernels (grains), which are characterized by a significant degree of asymmetry, causes disturbances in the development of the root system [51] and subsequently can affect the plant as a whole.

The contribution of climate change and increased effects of environmental stressors have had and will continue to affect the development and productivity of plants. The need for a transition to the digitalization of agriculture, especially for such a fundamental crop as wheat, is very urgent [52,53]. Therefore, identifying the effects of each individual factor is critical to the development of projected grain production. By analyzing the relationships between traits and environment conditions, 3D X-ray micro computed tomography (CT) images find that the grain-to-grain distance, aspect ratio, and porosity are more likely affected by the genome than environment [44].

Earlier, we made sure that the degree of manifestation of asymmetry indices is expressed differently in different varieties of wheat [7]. The phenomenon of peculiarities in the formation of right and left seeds is widespread. In the present work, we studied which parts of the kernel can be used to understand the structural basis for changes of ideal morphology. For this, an idealized model based on conditional cylinders, reflected in the diagram in the form of circles, was considered (Supplementary Material Figures S1 and S2). In cases where no differences in the shape and diameter of the circles from the idealized model were noted in the studied specimen, yellow coloring was used. Where significant deviations from the idealized model were noted, this is indicated in orange in those circles that had differences. Although some kernels could be considered symmetrical and corresponding to the idealized model, slight deformation in the region of the groove (crease) was observed. Thus, it was found that larger kernels were characterized by an increase in area due to an enlargement in the circle area in the upper part of the corresponding part of the kernels (Supplementary Material Figure S1). In asymmetric kernels of medium size, there was an increase in the area of the circle in the lower part of the kernel (Figure 3 and Supplementary Material Figure S2). Asymmetric kernels from the upper parts of the spikelets were smaller, but generally consistent with the idealized model.

Interestingly, in unfulfilled kernels, the asymmetry of the parts is preserved. We also raised the issue of comparing the asymmetry factor and kernel fulfillment. Asymmetry did not have a significant effect on the preservation of all components of the kernel fine structure in comparison with symmetrical kernels. While a looser distribution of starch grains was observed in unfulfilled kernels, non-binding components of the cytoplasm and the formation of cavities were observed. Such a structure improves the access of oxygen to internal structures and, accordingly, makes it problematic and very limited to use accelerated selection using immature grains, since their storage even for a short time is unlikely to be possible due to the rapid loss of germination.

In this paper, we expected to see the negative effect of salinity, a priori assuming that salinity is undesirable, as widely reported in long-term studies [11,54,55]. However, it was found that the characteristics of the kernels studied were rather good than bad. Meanwhile, it is worth considering the probable causes of the noted effect, which should be taken into account when looking for ways to reduce the negative effects of salinity, usually mitigated by the use of salt-tolerant varieties and the application of specific fertilizers and agrichemicals [56]. A possible reason could be that the salt concentration was not high enough to cause a noticeable drop in yield. In addition, the influence of salt ions and their interaction with plant roots and the soil-absorbing complex could cause the priming effect, i.e., the triggering of a cascade of protective reactions in plants, leading to their better adaptation to the high temperatures and drought characteristic of this region. It is also necessary to take into account the soil and root-associated microflora, which, with the addition of NaCl, could be modified in a way that provides benefits for the growth and development of the root system in this particular case.

This study of the differences in wheat kernel will be greatly helpful for accelerated breeding if we can automatically discover wheat phenotype in a nondestructive and fast manner in perspective [44]. Salinity, as a damaging factor that causes oxidative, osmotic, and toxic effects, is a rather complex stress. Further experiments will likely require simplifying the models inducing osmotic and oxidative stresses, for example using paraquat or hydrogen peroxide to trigger oxidative effects [57] and PEG to simulate drought [58].

## 4. Materials and Methods

### 4.1. Plant Material

Materials for the study were obtained from the Federal State Budgetary Scientific Institution “Federal Scientific Center for Biological Systems and Agrotechnologies of the Russian Academy of Sciences” (Orenburg).

The following varieties of wheat were taken as objects of study:

- Zolotaya—spring hard wheat, a variety of hordeiforme;
- Orenburgskaya 10—spring hard wheat, a variety of hordeiforme;
- Orenburgskaya 23—soft spring wheat, Lutescens variety;
- Ulyanovskaya 105—soft spring wheat, Lutescens variety.

The experiment was carried out in the central zone of the Orenburg region (fields coordinates—51°46′41.928000″ N, 55°19′14.292000″ E, 51°46′43.074000″ N, 55°19′14.598000″ E, 51°46′43.338000″ N, 55°19′22.620000″ E 51°46′42.120000″ N, 55°19′22.404000″ E).

The soil is southern carbonate low-humus heavy loamy chernozem, pH of the soil solution is 7.0–8.0 (pH meter brand—Ecotest, Moscow, Russia).

Sowing varieties were carried out on a black fallow. Site preparation consisted of non-moldboard loosening after harvesting the previous crop, early spring harrowing, four-fold cultivation during the summer, and autumn deep loosening of the fallow. In the year of sowing the experiment, spring harrowing of the site and pre-sowing cultivation to the depth of seeding were carried out.

Sowing was carried out using a seeder SN-16 in an ordinary way to a depth of 6–8 cm with a seeding rate of 4.5 million seeds per ha, followed by post-sowing rolling with ring-spur rollers. The area of plots under each variety was 50 m^2^, each variety was sown in three repetitions (Supplementary Materials Figure S3).

To start the experiment on salt stress, in two non-adjacent repetitions for each variety, microplots of 0.50 m^2^ were selected, which included three rows of sowing with a row spacing of 15 cm with a row length of 111 cm. The total number of microplots in the experiment for each variety was 30 (Supplementary Materials Figure S3).

For watering, the solutions of two salts, NaCI and Na_2_SO_4_, were used. Salt solutions were prepared by dissolving NaCl in water at a concentration of 1.10 g L^−1^, and Na_2_SO_4_ at a concentration of 0.40 g L^−1^. The plots were irrigated once, in the phase of full shoots. The control was irrigated with water, simultaneously with the watering of the experimental variants. The water consumption rate was 30 L per m^2^, which corresponds to the monthly rainfall rate in the region. After watering, the plots were mulched with dry soil.

In the tillering phase, 10 plants were selected for each wheat cultivar to analyze the root system of wheat plants. In the earing phase, 10 plants of each wheat cultivar were selected for accounting the height of the plants and the length of the spike.

In the phase of full maturing of the wheat grain in the plots, 5 spikes were randomly selected, the kernels of which were used for further analysis (Supplementary Materials Figure S4).

### 4.2. Preparation of Material for Primary Analysis

All available spikes were disassembled into simple ones, keeping their placement order on the main spike axis. Next, the kernels were extracted and glued due to double-sided tape onto a white sheet of A4 paper while maintaining the order of the kernels in the spike. Each sheet of paper contained kernels of only one cultivar with a specific salt exposure variant. Images were scanned by a professional Epson Perfection V550 Photo scanner with a resolution of 600 dpi. The resulting images were processed in the ImageJ program [59]. The transverse section area (mm^2^), transverse section perimeter (mm), length (mm), and width (mm) of kernels were measured by the program. Then, the mass of each kernel was measured on electronic scales (*p* = 0.0001 g). In total, 1173 kernels were examined in the study (Supplementary Material Table S1).

A sufficient sample size was collected to compare fluctuating asymmetry indices according to the algorithm proposed earlier for horticultural crops [60].

It was assumed that the volume of the general population of kernels tends to infinity $\hat{N}\to \infty$, therefore, Student’s criterion was taken as t_st_05 = 1.96. It was assumed that the minimum value of the asymmetry index in general population (x_min_) can be equal to zero, which corresponds to a completely symmetrical kernel, and the maximum value (x_max_) is approx. 0.2 (based on the information on the fluctuating asymmetry scale proposed earlier [7]. Using the 6-sigma rule, the value of the standard deviation $\hat{\sigma }$ was determined:

$$\hat{\sigma }=(x_{\max}-x_{\min})/6=(0.2-0)/6=0.333$$

Allowable error ∆ = 0.035 (corresponds to the class interval on the fluctuating asymmetry scale) or ∆ = 0.017 (corresponds to half of the class interval on the fluctuating asymmetry scale) proposed earlier [7].

Allowable accuracy (*k*) was calculated by the following formula:

$$k=\frac{\Delta }{\hat{\sigma }}$$

*k* = 0.035/0.333 = 1.05 or *k* = 0.017/0.333 = 0.51

The sample size (N) was calculated using the following formula:

N = t_st_^2^/*k*^2^ (1)

N = (1.96 × 1.96)/(1.05 × 1.05) = 3.69, the minimum sample size for a sample to obtain a reliable value is 4 kernels.

Or

N = (1.96 × 1.96)/(0.51 × 0.51) = 14.77, i.e., this ideal sample size, minimizing the experimental error, is 15 kernels.

After data on kernel parameters were entered into the SPSS Statistics 25 program, the “random number generator” function was turned on and the SPSS program randomly selected 15 matured left, right, and middle kernels for each of the four varieties in three salinity options (the algorithm is given in the Supplementary Material Table S1 about the number of kernels) where the number of mature kernels exceeded 15 pieces. If the number of mature kernels was less than 15, for example, 6 pieces (Zolotaya, control, middle from the Supplementary Material Table S1), then all 6 kernels were taken.

**Figure 4:**
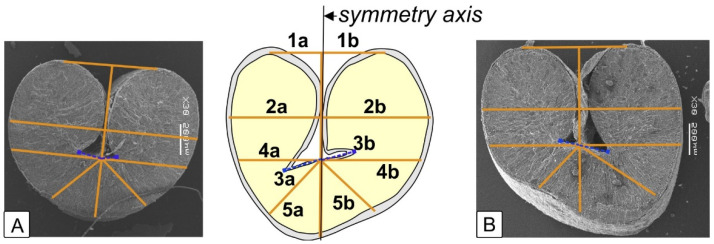
Distances between the control points of the left and right sides of the kernel to the axis of symmetry. Fluctuating asymmetry indices: 1a,b—arm lengths from the intersection of the perpendicular to the tangent (axis of symmetry) to the shares of halves of the wheat kernel on the median section; 2a,b—arm lengths of the perpendicular to the widest sections of the shares of halves of the wheat kernel; 3a,b—segment lengths of maximum distance of the inner corners of the crease (ventral furrow) from the intersection point of the symmetry axis with the flat part of the ventral furrow between the halves of the shares of wheat kernel; from 4a,b—the arm lengths of the perpendicular at the point of intersection of the symmetry axis with the flat part of the ventral furrow between the halves of the wheat kernel shares; 5a,b—the lengths of the bisectors extending from the perpendicular at the point of intersection of the symmetry axis with the flat part of the ventral furrow between the halves of the wheat kernel shares.

458 transverse slices of kernels were prepared, of which 113 sections were from the cv Zolotaya, 93 sections were from the cv Orenburgskaya 10, 125 sections were from the cv Orenburgskaya 23, and 127 sections were from the cv Ulyanovskaya 105. Sections were made from the middle part of the kernels. The sections were glued with double-sided adhesive tape on a white A4 sheet; then, the images were scanned on an Epson Perfection V550 Photo with a resolution of 600 dpi. In the ImageJ program by means of the “segmented line” tool the length of the central axis of the kernel cut, the distance from the control points of the left and right sides of the kernel to the axis to establish asymmetry indices were measured, in millimeters. Index 1 is the ratio of the distance from the top of the kernel to the central axis to the total distance between the tops. Index 2 is the ratio of the distance along the widest part to the central axis to the total distance of width. Index 3 is the ratio of the distance from the edge of the kernel under the triangle to the central axis to the total distance. Index 4 is the ratio of the distance from the edge of the triangle to the axis of symmetry to the total length of the triangle. Index 5 is the ratio of each segment extending 45° from the axis of symmetry to the length segment from the triangle to the bottom (Figure 4).

Fluctuating asymmetry (FA) indices were calculated by the formula of Wilsey et al. (1998) [30].

$$\mathrm{FA}=\frac{|L_{1}-L_{1}^{\prime }|}{L_{1}+L_{1}^{\prime }}+\frac{|L_{2}-L_{2}^{\prime }|}{L_{2}+L_{2}^{\prime }}$$

L_1_ corresponds to the length from the left top of the kernel to the axis of symmetry, and L_1_′ corresponds to the length from the right top of the kernel to the axis of symmetry. The measurements L_1_ and L_1_′ are obtained for calculating index 1 (described above), L_2_ and L_2_′ for index 2, and so on.

All statistic calculations were obtained by IBM SPSS Statistics 25 program. Two-way ANOVA was used to determine the effect of salts on embryonic, adventitious, and nodal roots, as well as on plant height and spike length. The difference between the variants was determined by LSD (least significant difference test). The compliance of the sample with the normal distribution law was verified using Kolmogorov–Smirnov method. Since the data in most cases did not conform the normal distribution law, despite the significant sampling size, Kruskal–Wallis analysis of variance for non-parametric criteria (*p* = 0.05) was used to establish differences between the experimental variants. This method allows us to check the statement that salt exposure affects the parameters of wheat kernels. The principal component method was used to decrease the dimension.

## 5. Conclusions

A slight salt effect of NaCl and Na_2_SO_4_ on wheat plants of four cultivars caused an increase in the weight of kernels, as well as a number of other parameters of kernel (perimeter of transverse section, area of transverse section, length, and width). Methods for assessing fluctuating asymmetry revealed a trend towards an increase in the number of symmetrical kernels in a spike. However, this trend was reliably confirmed only in the case of exposure to NaCl for the central kernels in the spikelet in the cultivars Zolotaya, Orenburgskaya 10, and Orenburgskaya 23. Additionally, similar results were obtained for the central kernels in spikelets in the cv Zolotaya as a result of exposure to Na_2_SO_4_, and for the left kernels in spikelets in the cv Ulyanovskaya 105 exposed to NaCl. Thus, the methods proposed in this research for assessing fluctuating asymmetry in wheat kernels under field conditions were able to reliably confirm the observed deviations as a result of exposure to low salt concentrations not for all the studied specimens. It is possible that when using the proposed indices for assessing fluctuating asymmetry at high salt concentrations corresponding to natural salt stress, both an increase and a decrease in asymmetry can be detected, which requires further study.

## Figures and Tables

**Table 1:** Characteristics of the root system of spring wheat cultivars under salt stress caused by different salts.

Cultivar	Experiment Variant	Embryonic Roots		Adventitious Roots, pcs.	Nodal Roots, pcs.
		Number, pcs.	Length, cm		
Orenburgskaya 23	Control	4.4 ± 1.4	5.2 ± 3.0	1.5 ± 0.7	0.7 ± 0.3
	NaCl—1.1 g L^−1^	3.3 ± 1.4	4.5 ± 3.1	2.2 ± 0.6	1.5 ± 0.6
	Na_2_SO_4_—0.40 g L^−1^	3.9 ± 1.0	4.0 ± 2.1	1.9 ± 0.3	1.0 ± 0.4
Ulyanovskaya 105	control	4.7 ± 1.2	4.3 ± 2.0	5.0 ± 2.3	3.8 ± 1.3
	NaCl—1.1 g L^−1^	3.9 ± 1.2	5.1 ± 3.3	2.6 ± 1.4	2.8 ± 1.5
	Na_2_SO_4_—0.40 g L^−1^	4.0 ± 1.6	4.8 ± 2.5	2.5 ± 1.1	1.7 ± 0.7
Orenburgskaya 10	control	4.8 ± 1.8	3.5 ± 1.7	5.4 ± 2.0	1.3 ± 0.5
	NaCl—1.1 g L^−1^	4.7 ± 0.9	4.1 ± 2.0	2.8 ± 1.3	0.9 ± 0.3
	Na_2_SO_4_—0.40 g L^−1^	5.9 ± 1.6	4.9 ± 2.2	4.7 ± 2.5	2.5 ± 1.2
Zolotaya	control	5.1 ± 1.3	5.5 ± 2.3	4.2 ± 2.0	1.7 ± 0.7
	NaCl—1.1 g L^−1^	4.2 ± 0.8	4.4 ± 2.6	2.3 ± 0.5	0.8 ± 0.3
	Na_2_SO_4_—0.40 g L^−1^	3.9 ± 1.2	5.2 ± 2.9	4.8 ± 0.6	1.6 ± 0.6
LSD _05_		0.62	0.08	0.67	0.72
LSD _05_A (Cultivar)		0.51	0.74	0.55	0.53
LSD _05_B (Experiment variant)		0.44	0.62	0.48	0.42
LSD _05_AB		0.44	0.62	0.48	0.42

LSD—least significant difference test (*p* = 0.05). Arithmetic mean ± standard deviation, *p* = 0.05.

**Table 2:** Influence of different salinity variants on the morphological parameters of spring wheat cultivars in the earing phase.

Cultivar	Experiment Variant	Plant Height, cm	Spike Length, cm
Orenburgskaya 23	control	46.7 ± 6.8	8.4 ± 0.6
	NaCl—1.1 g L^−1^	49.1 ± 6.2	6.5 ± 0.7
	Na_2_SO_4_—0.40 g L^−1^	43.1 ± 6.5	8.0 ± 1.1
Ulyanovskaya 105	control	55.6 ± 10.4	6.8 ± 0.9
	NaCl—1.1 g L^−1^	57.6 ± 8.3	6.2 ± 1.4
	Na_2_SO_4_ − 0.40 g L^−1^	51.0 ± 6.9	7.6 ± 0.9
Orenburgskaya 10	control	60.0 ± 9.9	6.6 ± 0.7
	NaCl—1.1 g L^−1^	52.0 ± 7.9	5.3 ± 0.9
	Na_2_SO_4—_0.40 g L^−1^	47.0 ± 6.6	5.8 ± 0.8
Zolotaya	control	61.6 ± 6.0	6.8 ± 0.6
	NaCl—1.1 g L^−1^	63.4 ± 10.5	7.8 ± 0.8
	Na_2_SO_4—_0.40 g L^−1^	62.0 ± 9.8	6.6 ± 1.0
LSD _05_		3.82	0.41
LSD _05_A (Cultivar)		3.12	0.33
LSD _05_B (Experiment variant)		2.70	0.29
LSD _05_AB		2.70	0.29

LSD—least significant difference test (*p* = 0.05). Arithmetic mean ± standard deviation, *p* = 0.05.

**Table 3:** Confirmation of the hypothesis about the significant effect of salts on the parameters of kernels in different wheat cultivars.

	Kernel Weight	Area	Perimeter	Length	Width
Orenburgskaya 10	–	+	+	+	+
Zolotaya	+	+	+	–	+
Ulyanovskaya 105	+	+	+	+	+
Orenburgskaya 23	–	–	+	–	–

Confirmation was made by the Kruskal–Wallis analysis of variance (*p* = 0.05), where “+” there is influence, “–“ no influence.

**Table 4:** Average values of the general (fluctuating) asymmetry of kernels in wheat cultivars with different salt exposure variants.

	Control	NaCl	Na_2_SO_4_
Zolotaya	0.140 ± 0.055	0.111 ± 0.047	0.095 ± 0.05
Orenburgskaya 10	0.115 ± 0.009	0.109 ± 0.009	0.134 ± 0.011
Ulyanovskaya 105	0.109 ± 0.008	0.106 ± 0.007	0.107 ± 0.007
Orenburgskaya 23	0.111 ± 0.009	0.099 ± 0.008	0.097 ± 0.008

Arithmetic mean ± standard deviation, *p* = 0.05.

**Table 5:** Scale of indicators of overall asymmetry.

Asymmetry Range	Degree of Asymmetry
0.010–0.070	Very low
0.071–0.131	Low
0.132–0.192	Middle
0.193–0.253	High
0.254–0.314	Very high

The arithmetic means were calculated for five asymmetry indices in 739 kernels of cv Zlata, Agata, Rubezhnaya, Ulyanovskaya 105, Orenburgskaya 10, Orenburgskaya 23, Zolotaya, and average asymmetry values obtained were divided into 5 classes manifesting the degree of asymmetry.

**Table 6:** Distribution of the number of kernels in the sampling according to the degree of asymmetry.

Cultivar	Salinity Variant	Degree of Fluctuating Asymmetry					Total Kernels, Pieces
		Very Low	Low	Middle	High	Very High	
Zolotaya	Control	3	15	10	6	2	36
	NaCl	9	16	11	1	0	37
	Na_2_SO_4_	15	18	5	2	0	40
Total kernels, pieces		**27**	**49**	**26**	**9**	**2**	**113**
Orenburgskaya 10	Control	6	13	9	3	0	31
	NaCl	11	12	9	1	1	34
	Na_2_SO_4_	3	14	4	5	2	28
Total kernels, pieces		**20**	**39**	**22**	**9**	**3**	**93**
Ulyanovskaya105	Control	10	18	9	3	1	41
	NaCl	7	24	7	4	0	42
	Na_2_SO_4_	7	25	9	3	0	44
Total kernels, pieces		**24**	**67**	**25**	**10**	**1**	**127**
Orenburgskaya 23	Control	10	20	10	3	1	44
	NaCl	13	21	6	1	3	44
	Na_2_SO_4_	16	14	3	4	0	37
Total kernels, pieces		**39**	**55**	**19**	**8**	**4**	**125**

Bold type indicates the numbers corresponding to the total number of kernels in the experiment variant.

## Data Availability

Not applicable.

## Supplementary Materials

The following supporting information can be downloaded at: https://www.mdpi.com/article/10.3390/plants12050980/s1, Figure S1: Scanning electron microscopy of transverse sections of the central part of wheat kernels with manifested asymmetry; Figure S2: Scanning electron microscopy of transverse sections of the central part of wheat kernels with different degrees of fulfillment; Figure S3: Wheat plants with root system, experimental plots, scheme of experiment; Figure S4: Appearance of mature spikes of wheat; Table S1: The number of kernels involved in the experiment.

## References

[1] Afzal I., Basra S.M.A., Hameed A., Farooq M. (2006). Physiological enhancements for alleviation of salt stress in wheat. Pak. J. Bot..

[2] Koppolu R., Chen S., Schnurbusch T. (2022). Evolution of inflorescence branch modifications in cereal crops. Curr. Opin. Plant Biol..

[3] Frølich W., Åman P. (2010). Whole grain for whom and why?. Food Nutr. Res..

[4] Chaban I.A., Gulevich A.A., Smirnova E.A., Baranova E.N. (2021). Morphological and ultrastructural features of formation of the skin of wheat (*Triticum aestivum* L.) kernel. Plants.

[5] Fischer C., Neuhaus G. (1996). Influence of auxin on the establishment of bilateral symmetry in monocots. Plant J..

[6] Zhou H., Riche A.B., Hawkesford M.J., Whalley W.R., Atkinson B.S., Sturrock C.J., Mooney S.J. (2021). Determination of wheat spike and spikelet architecture and grain traits using X-ray Computed Tomography imaging. Plant Methods.

[7] Baranova E.N., Aniskina T.S., Kryuchkova V.A., Shchuklina O.A., Khaliluev M.R., Gulevich A.A. (2022). Evaluation of the heterogeneity of wheat kernels as a traditional model object in connection with the asymmetry of development. Symmetry.

[8] Boshnakian S. (1918). The mechanical factors determining the shape of the wheat kernel. J. Amer. Soc. Agron..

[9] Shewry P.R., Evers A.D., Bechtel D.B., Abecassis J., Khan K., Shewry P. (2009). Development, structure, and mechanical properties of the wheat grain. Wheat Chemistry and Technology.

[10] Cossani C.M., Sadras V.O. (2021). Symmetric response to competition in binary mixtures of cultivars associates with genetic gain in wheat yield. Evol. Appl..

[11] El Sabagh A., Islam M.S., Skalicky M., Ali Raza M., Singh K., Anwar Hossain M., Hossain A., Mahboob W., Iqbal M.A., Ratnasekara D. (2021). Salinity stress in wheat (*Triticum aestivum* L.) in the changing climate: Adaptation and management strategies. Front. Agron..

[12] Rengasamy P. (2006). World salinization with emphasis on Australia. J. Exp. Bot..

[13] Kaushal S.S., Groffman P.M., Likens G.E., Belt K.T., Stack W.P., Kelly V.R., Band L.E., Fisher G.T. (2005). Increased salinization of fresh water in the northeastern United States. Proc. Natl. Acad. Sci. USA.

[14] Löfgren S. (2001). The chemical effects of deicing salt on soil and stream water of five catchments in southeast Sweden. Water Air Soil Pollut..

[15] Manca F., Capelli G., Tuccimei P. (2015). Sea salt aerosol groundwater salinization in the Litorale Romano natural reserve (Rome, Central Italy). Environ. Earth Sci..

[16] Chamberlain S.D., Hemes K.S., Eichelmann E., Szutu D.J., Verfaillie J.G., Baldocchi D.D. (2020). Effect of drought-induced salinization on wetland methane emissions, gross ecosystem productivity, and their interactions. Ecosystems.

[17] Abuduwaili J., Liu D.W., Wu G.Y. (2010). Saline dust storms and their ecological impacts in arid regions. J. Arid Land.

[18] Herbert E.R., Boon P., Burgin A.J., Neubauer S.C., Franklin R.B., Ardón M., Hopfensperger K.N., Lamers L.P.M., Gell P. (2015). A global perspective on wetland salinization: Ecological consequences of a growing threat to freshwater wetlands. Ecosphere.

[19] Zeeshan M., Lu M., Sehar S., Holford P., Wu F. (2020). Comparison of biochemical, anatomical, morphological, and physiological responses to salinity stress in wheat and barley genotypes deferring in salinity tolerance. Agronomy.

[20] Kononenko N., Baranova E., Dilovarova T., Akanov E., Fedoreyeva L. (2020). Oxidative damage to various root and shoot tissues of durum and soft wheat seedlings during salinity. Agriculture.

[21] Al-Ashkar I., Alderfasi A., Ben Romdhane W., Seleiman M.F., El-Said R.A., Al-Doss A. (2020). Morphological and genetic diversity within salt tolerance detection in eighteen wheat genotypes. Plants.

[22] Semenova G., Fomina I., Ivanov A. (2014). Combined effect of water deficit and salt stress on the structure of mesophyll cells in wheat seedlings. CellBio.

[23] Aldesuquy H.S. (2015). Impact of seawater salinity on ultrastructure of chloroplasts and oleosomes in relation to fat metabolism in flag leaf of two wheat cultivars during grain-filling. Adv. Crop Sci. Technol..

[24] Radi A.A., Farghaly F.A., Hamada A.M. (2013). Physiological and biochemical responses of salt-tolerant and salt-sensitive wheat and bean cultivars to salinity. J. Biol. Earth Sci..

[25] Zhao D., Gao S., Zhang X., Zhang Z., Zheng H., Rong K., Zhao W., Khan S.A. (2021). Impact of saline stress on the uptake of various macro and micronutrients and their associations with plant biomass and root traits in wheat. Plant Soil Environ..

[26] Quan X., Liang X., Li H., Xie C., He W., Qin Y. (2021). Identification and characterization of wheat germplasm for salt tolerance. Plants.

[27] Hódar J.A. (2002). Leaf fluctuating asymmetry of Holm oak in response to drought under contrasting climatic conditions. J. Arid Environ..

[28] Hopton M.E., Cameron G.N., Cramer M.J., Polak M., Uetz G.W. (2009). Live animal radiography to measure developmental instability in populations of small mammals after a natural disaster. Ecol. Indic..

[29] Kozlov M.V., Wilsey B., Koricheva J., Haukioja E. (1996). Fluctuating asymmetry of birch leaves increase under pollution impact. J. Appl. Ecol..

[30] Hagen S.B., Ims R.A., Yoccoz N.G., Sørlibråten O. (2008). Fluctuating asymmetry as an indicator of elevation stress and distribution limits in mountain birch (*Betula pubescens*). Plant Ecol..

[31] Lappalainen J.H., Martel J., Lempa K., Wilsey B., Ossipov V. (2000). Effects of resource availability on carbon allocation and developmental instability in cloned birch seedings. Int. J. Plant Sci..

[32] Wilsey B., Haukioja E., Koricheva J., Sulkinoja M. (1998). Leaf fluctuating asymmetry increases with hybridization and elevation in three-line birches. Ecology.

[33] Freeman D.C., Graham J.H., Tracy M., Emlen J.M., Alados C.L. (1999). Developmental instability as a means of assessing stress in plants: A case study using electromagnetic fields and soybeans. Int. J. Plant Sci..

[34] Zvereva E.L., Kozlov M.V., Niemela P., Haukioja E. (1997). Delayed induced resistance and increase in leaf fluctuating asymmetry as responses of *Salix borealis* to insect herbivory. Oecologia.

[35] Sherry R.A., Lord E.M. (1996). Developmental stability in leaves of *Clarkia tembloriensis* (*Onagraceae*) as related to population outcrossing rates and heterozygosity. Evolution.

[36] Souza G.M., Viana J.O.F., Oliveira R.F. (2005). Asymmetrical leaves induced by water deficit show asymmetric photosynthesis in common bean. Braz. J. Plant Physiol..

[37] Midgley G.F., Wand S.J.E., Musil C.F. (2002). Repeated exposure to enhanced UV-B radiation in successive generations increases developmental instability (leaf fluctuating asymmetry) in a desert annual. Plant Cell Environ..

[38] Milligan J.R., Krebs R.A., Mal T.K. (2008). Separating developmental and environmental effects on fluctuating asymmetry in *Lythrum salicaria* and *Penthorum sedoides*. Int. J. Plant Sci..

[39] Hochwender C.G., Fritz R.S. (1999). Fluctuating asymmetry in a *Salix hybrid* system: The importance of genetic versus environmental causes. Evolution.

[40] Valkama J., Kozlov M.V. (2001). Impact of climatic factors on the developmental stability of mountain birch growing in a contaminated area. J. Appl. Ecol..

[41] Anne P., Mawri F., Gladstone S., Freeman D.C. (1998). Is fluctuating asymmetry a reliable biomonitor of stress? A test using life history parameters in soybean. Int. J. Plant Sci..

[42] Rao G.-Y., Andersson S., Widen B. (2002). Flower and cotyledon asymmetry in *Brassica cretica*: Genetic variation and relationships with fitness. Evolution.

[43] Matilla A., Gallardo M., Puga-Hermida M.I. (2005). Structural, physiological and molecular aspects of heterogeneity in seeds: A review. Seed Sci. Res..

[44] Xiong B., Wang B., Xiong S., Lin C., Yuan X. (2019). 3D Morphological Processing for Wheat Spike Phenotypes Using Computed Tomography Images. Remote Sens..

[45] Faralli M., Williams K.S., Han J., Corke F.M., Doonan J.H., Kettlewell P.S. (2019). Water-saving traits can protect wheat grain number under progressive soil drying at the meiotic stage: A phenotyping approach. J. Plant Growth Regul..

[46] Shewry P.R., Wan Y., Hawkesford M.J., Tosi P. (2020). Spatial distribution of functional components in the starchy endosperm of wheat grains. J. Cereal Sci..

[47] Nuttall J.G., O’leary G.J., Panozzo J.F., Walker C.K., Barlow K.M., Fitzgerald G.J. (2017). Models of grain quality in wheat—A review. Field Crops Res..

[48] Mukhtarovna N.R., Kizi R.D.T., Kholdorovich A.K., Botiraliyevich U.N. (2021). Breathing of grain during storage and factors affecting the intensity of respiration. Int. Multidisc. Res. J..

[49] Bouchard J., Malalgoda M., Storsley J., Malunga L., Netticadan T., Thandapilly S.J. (2022). Health benefits of cereal grain-and pulse-derived proteins. Molecules.

[50] Chen X., Yin G., Börner A., Xin X., He J., Nagel M., Liu X., Lu X. (2018). Comparative physiology and proteomics of two wheat genotypes differing in seed storage tolerance. Plant Physiol. Biochem..

[51] Baranova E.N., Gulevich A.A. (2021). Asymmetry of plant cell divisions under salt stress. Symmetry.

[52] Sarkar A., Fu B.X. (2022). Impact of quality improvement and milling innovations on durum wheat and end products. Foods.

[53] Mondejar M.E., Avtar R., Diaz H.L.B., Dubey R.K., Esteban J., Gómez-Morales A., Hallam B., Mbungu N.T., Okolo C.C., Prasad K.A. (2021). Digitalization to achieve sustainable development goals: Steps towards a smart green planet. Sci. Total Environ..

[54] Abbas G., Saqib M., Rafique Q., Rahman A.U., Akhtar J., Haq M.A.U., Nasim M. (2013). Effect of salinity on grain yield and grain quality of wheat (*Triticum aestivum* L.). Pak. J. Bot..

[55] Price P.B., Parsons J. (1979). Distribution of lipids in embryonic axis, bran-endosperm, and hull fractions of hulless barley and hulless oat grain. J. Agric. Food Chem..

[56] Zheng Y., Xu X., Simmons M., Zhang C., Gao F., Li Z. (2010). Responses of physiological parameters, grain yield, and grain quality to foliar application of potassium nitrate in two contrasting winter wheat cultivars under salinity stress. J. Plant Nutr. Soil Sci..

[57] Angelova M.B., Pashova S.B., Spasova B.K., Vassilev S.V., Slokoska L.S. (2005). Oxidative stress response of filamentous fungi induced by hydrogen peroxide and paraquat. Mycol. Res..

[58] Dong B., Zheng X., Liu H., Able J.A., Yang H., Zhao H., Zhang M., Qiao Y., Wang Y., Liu M. (2017). Effects of drought stress on pollen sterility, grain yield, abscisic acid and protective enzymes in two winter wheat cultivars. Front. Plant Sci..

[59] Broeke J., Pérez J.M.M., Pascau J. (2015). Image Processing with ImageJ: Extract and Analyze Data from Complex Images with ImageJ, the World’s Leading Image Processing Tool.

[60] Isachkin A.B., Kryuchkova B.A. (2020). Algoritmi opredelenia dostatochnykh ob’emov vyborok (na primere sadovykh rastenii). Bulleten Glavnogo botanicheskogo sada.

