# Supplementary material for: Unexpected Effects of Sulfate and Sodium Chloride Application on Yield Qualitative Characteristics and Symmetry Indicators of Hard and Soft Wheat Kernels
Source: Plants (Basel). 2023 Feb 21;12(5):980. doi: 10.3390/plants12050980 (PMC10004740; doi:10.3390/plants12050980)
Supplement: Supplementary file 1 [file plants-12-00980-s001.zip › plants-2167141-supplementary.pdf]

Supplementary Materials

Fugure S1.

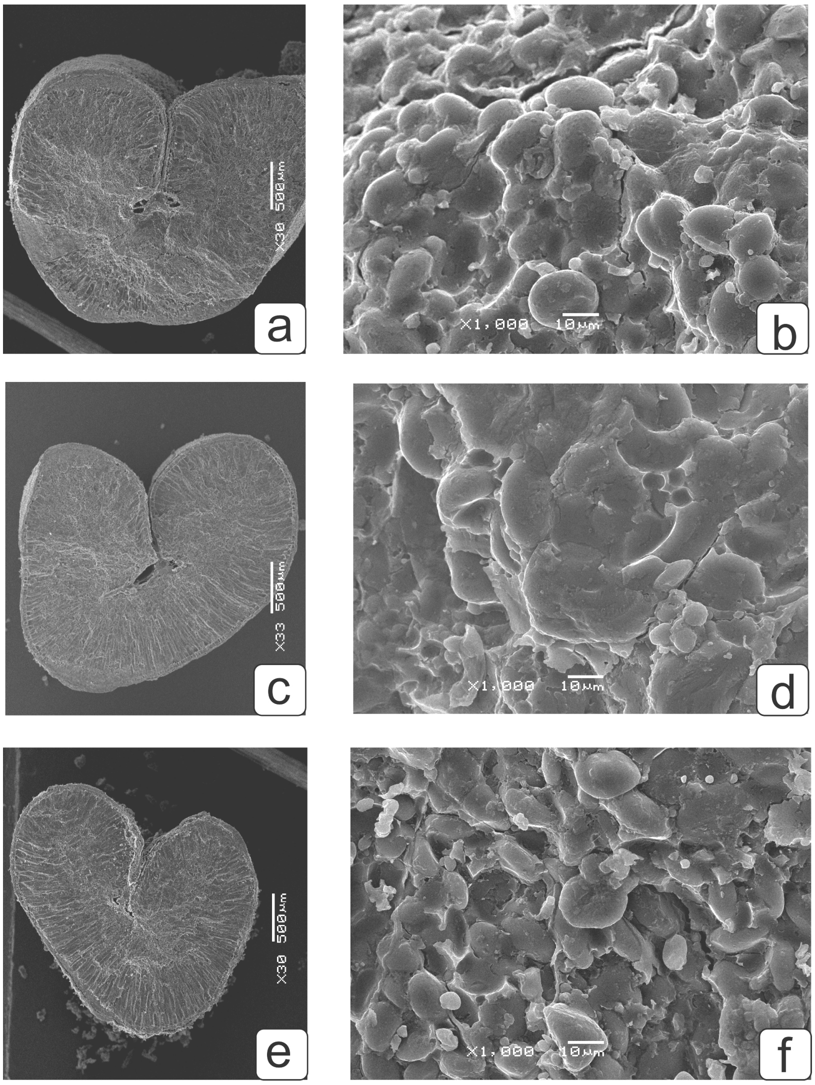

Figure S1. Scanning electron microscopy of transverse sections of the central part of wheat kernels with manifested asymmetry (a,c,e) and fragments of the central part of the right half of the corresponding sections: a,b - asymmetric kernel larger than average size, taken from the central part of the spike; c,d - asymmetric kernel of normal size, one of the two lower ones in the spikelet; e, f - asymmetric kernel of small size, one of the top two in the spikelet.

Figure S2.

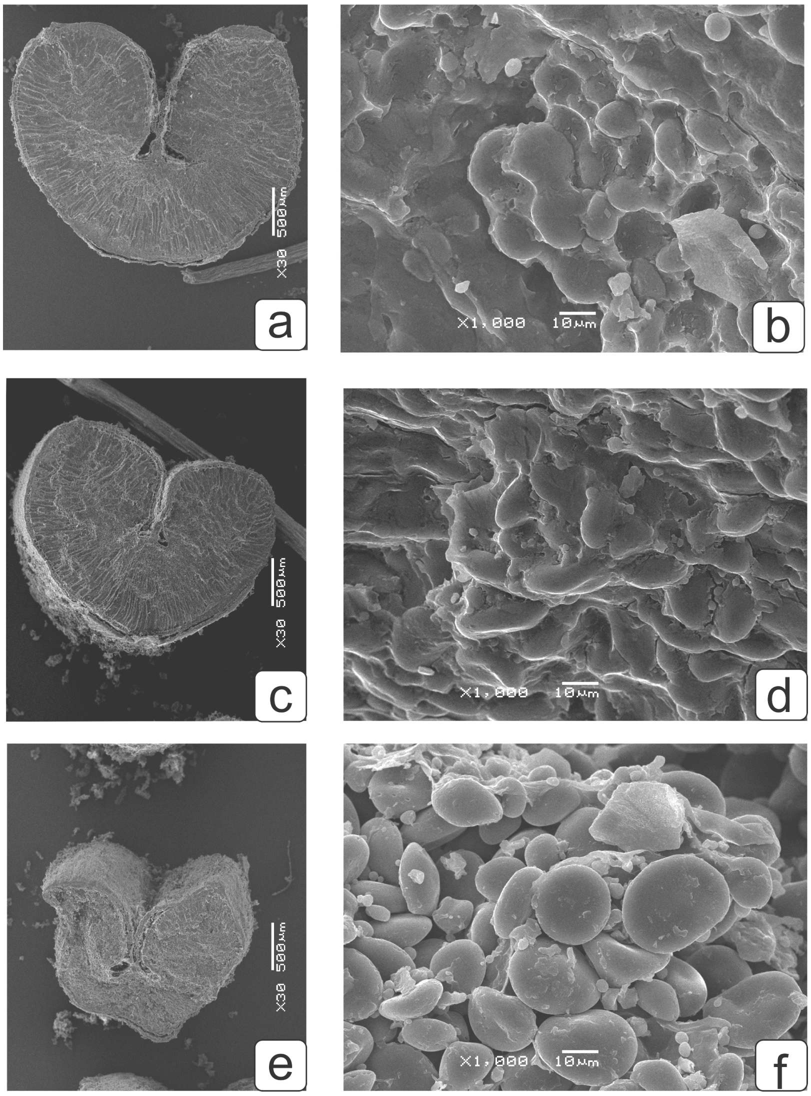

Figure S2. Scanning electron microscopy of transverse sections of the central part of wheat kernels (a,c,e) and fragments of the central part of the right half of the corresponding sections: a,b - relatively symmetrical kernel of medium size taken from the central part of the spike; c,d - relatively symmetrical small kernel, third in the spikelet; e,f - unfulfilled kernel, with manifested asymmetry.

Figure S3. Wheat plants with root system, Experimental plots, Scheme of experiment

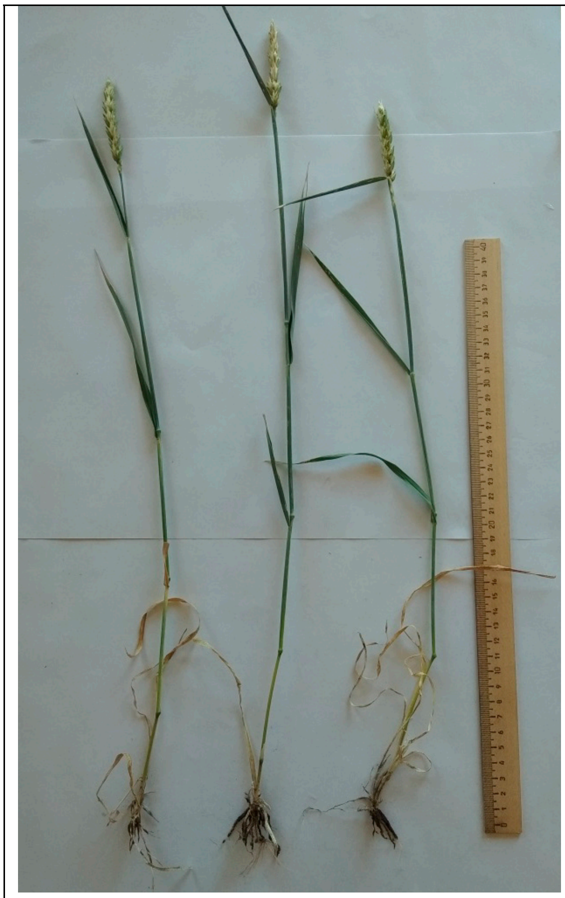	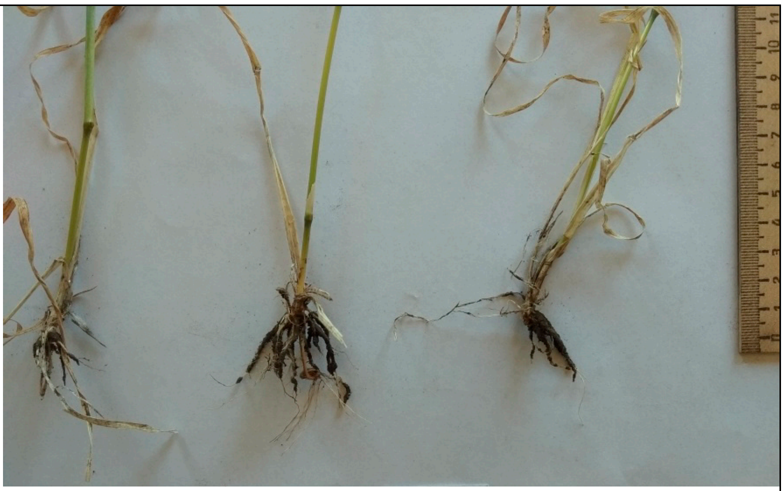
Orenburgskaya 23 (control). Milk stage.	Orenburgskaya 23 (control). Milk stage (roots).

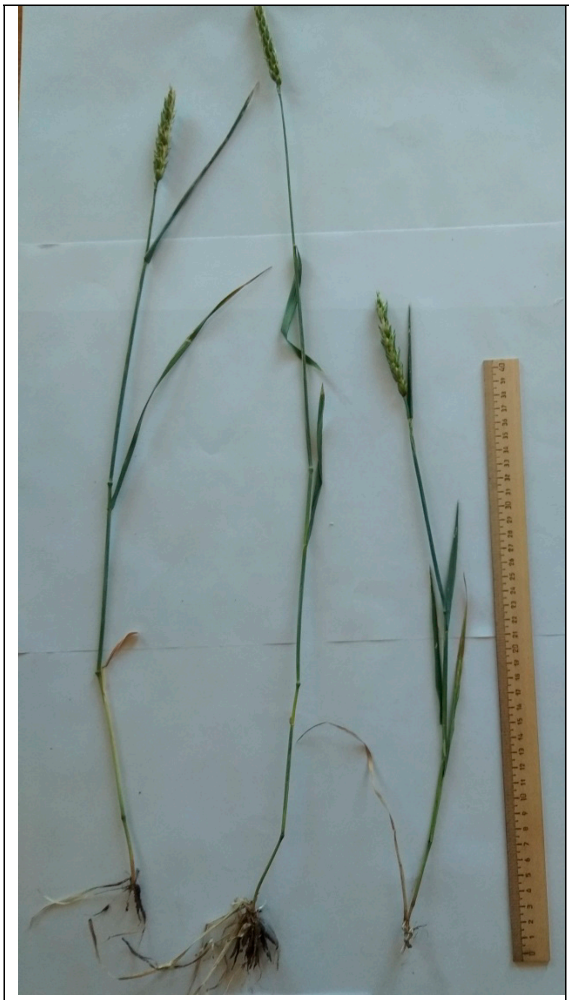	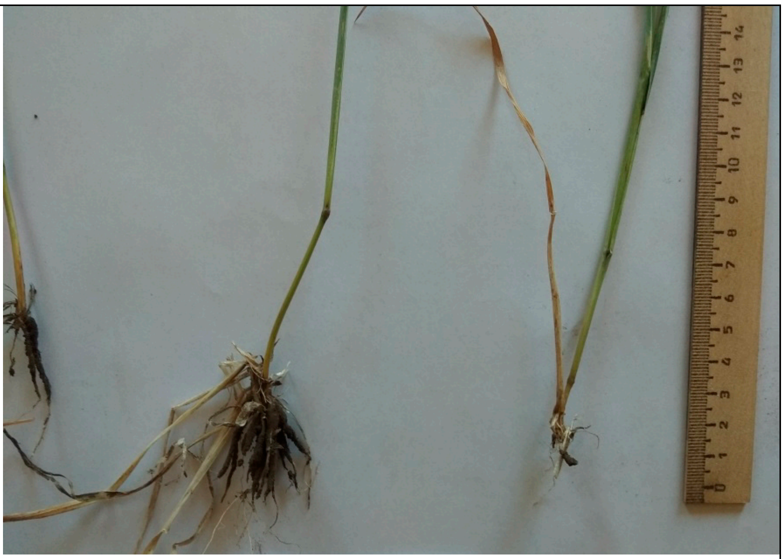
Orenburgskaya 23 (NaCl – 1.1 g L^{-1}). Milk stage.	Orenburgskaya 23 (NaCl – 1.1 g L^{-1}). Milk stage (roots).

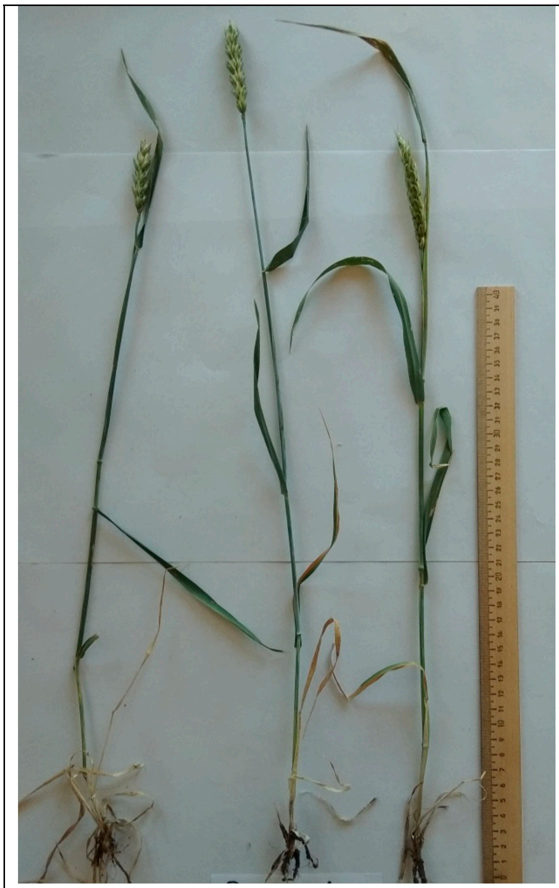	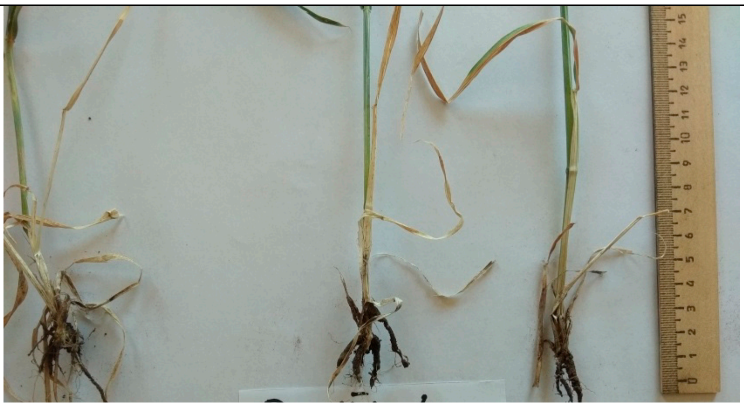
Orenburgskaya 23 (Na_2SO_4 – 0.4 g L^{-1}). Milk stage.	Orenburgskaya 23 (Na_2SO_4 – 0.4 g L^{-1}). Milk stage (roots).

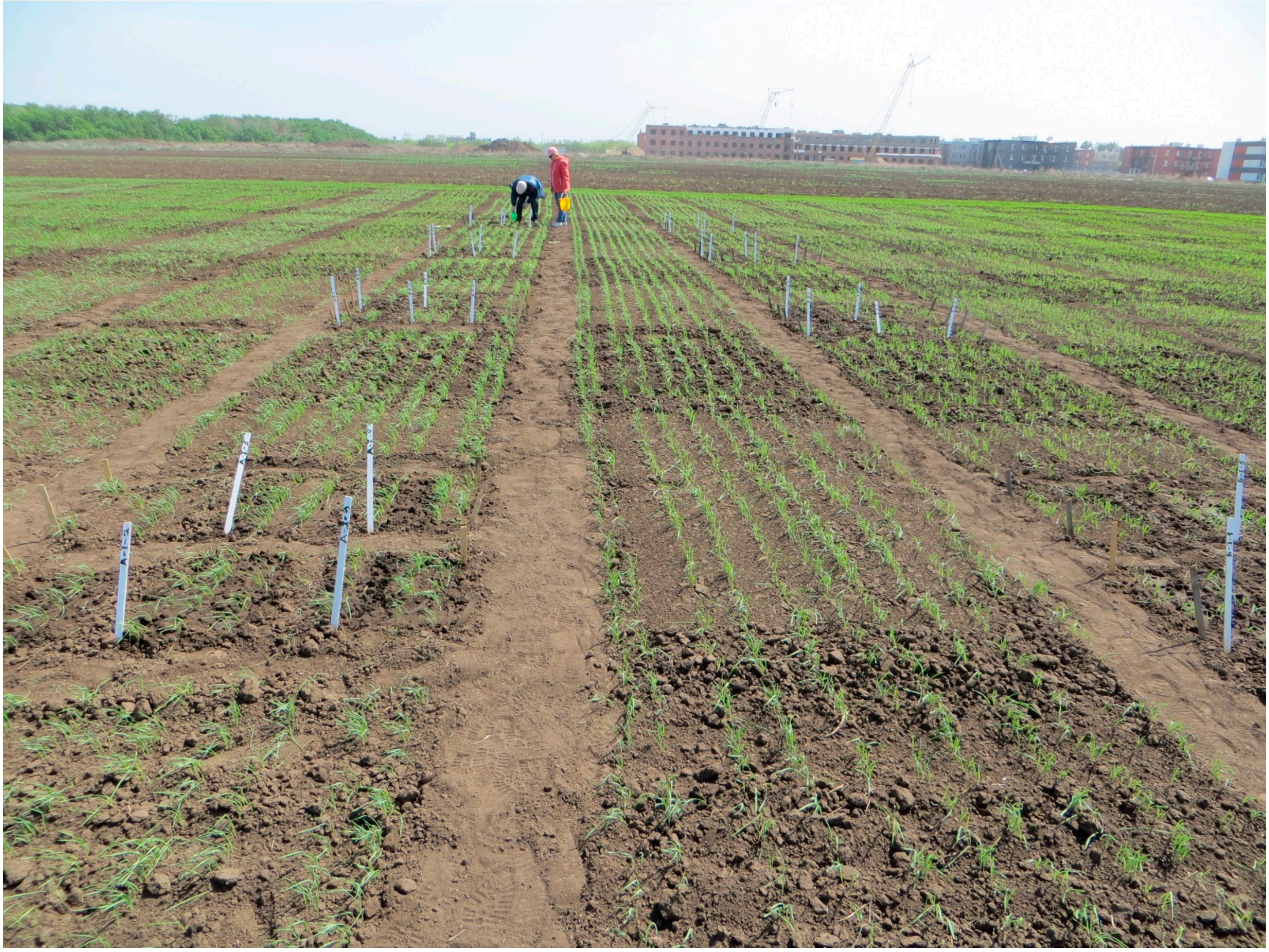

Experimental plots (general view)

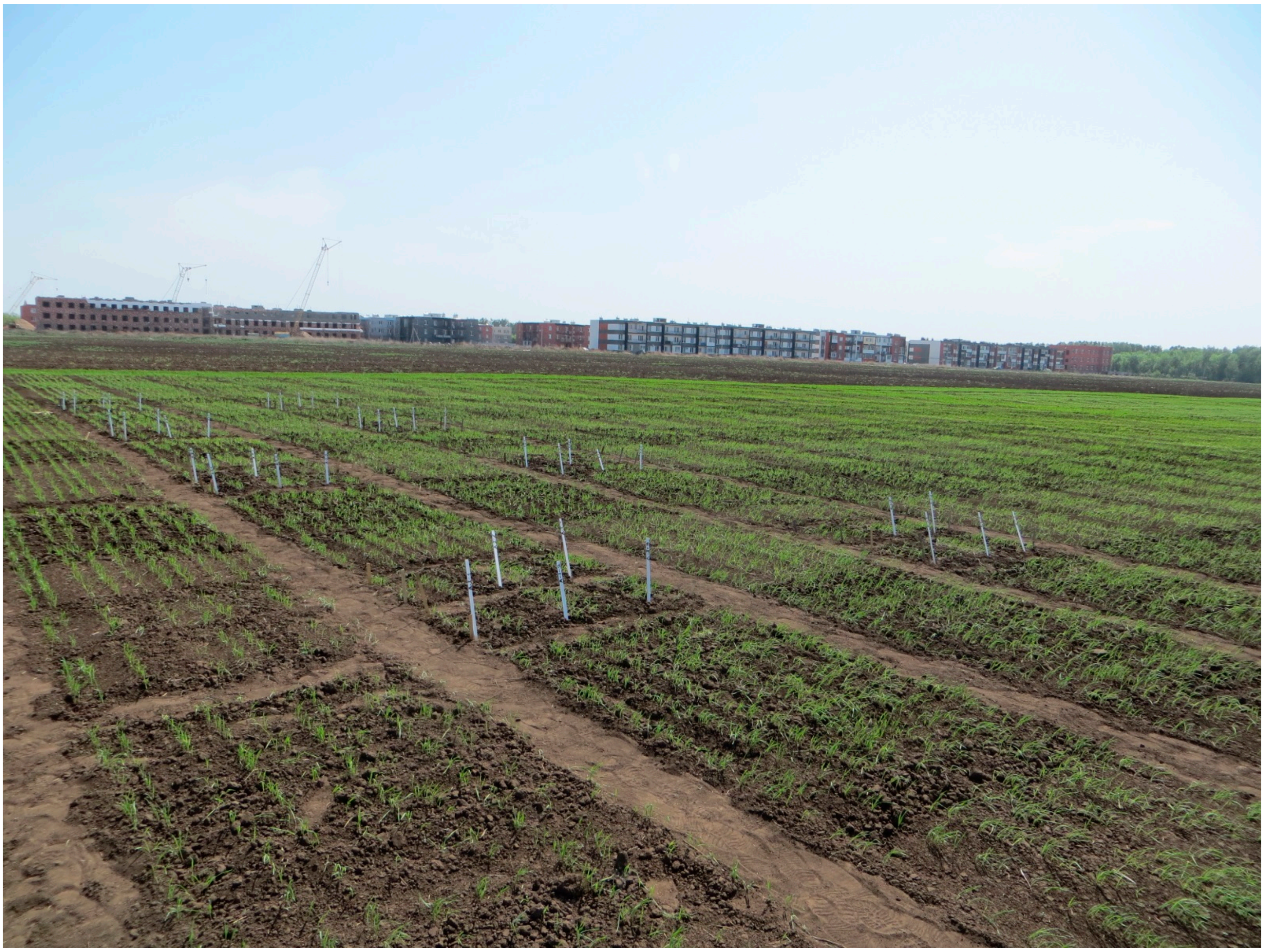

Experimental plots (general view)

Scheme of experience in one repeat

Cultivar – Orenburgskaya 23

Control	NaCl – 1.10 g L^{-1}	Na_2SO_4 – 0.4 g L^{-1}

Cultivar – Ulyanovskaya 105

Control	NaCl – 1.10 g L^{-1}	Na_2SO_4 – 0.4 g L^{-1}

Cultivar – Orenburgskaya 10

Control	NaCl – 1.10 g L^{-1}	Na_2SO_4 – 0.4 g L^{-1}

Cultivar – Zolotaya

Control	NaCl – 1.10 g L^{-1}	Na_2SO_4 – 0.4 g L^{-1}

Figure S4. Appearance of mature spikes of wheat.

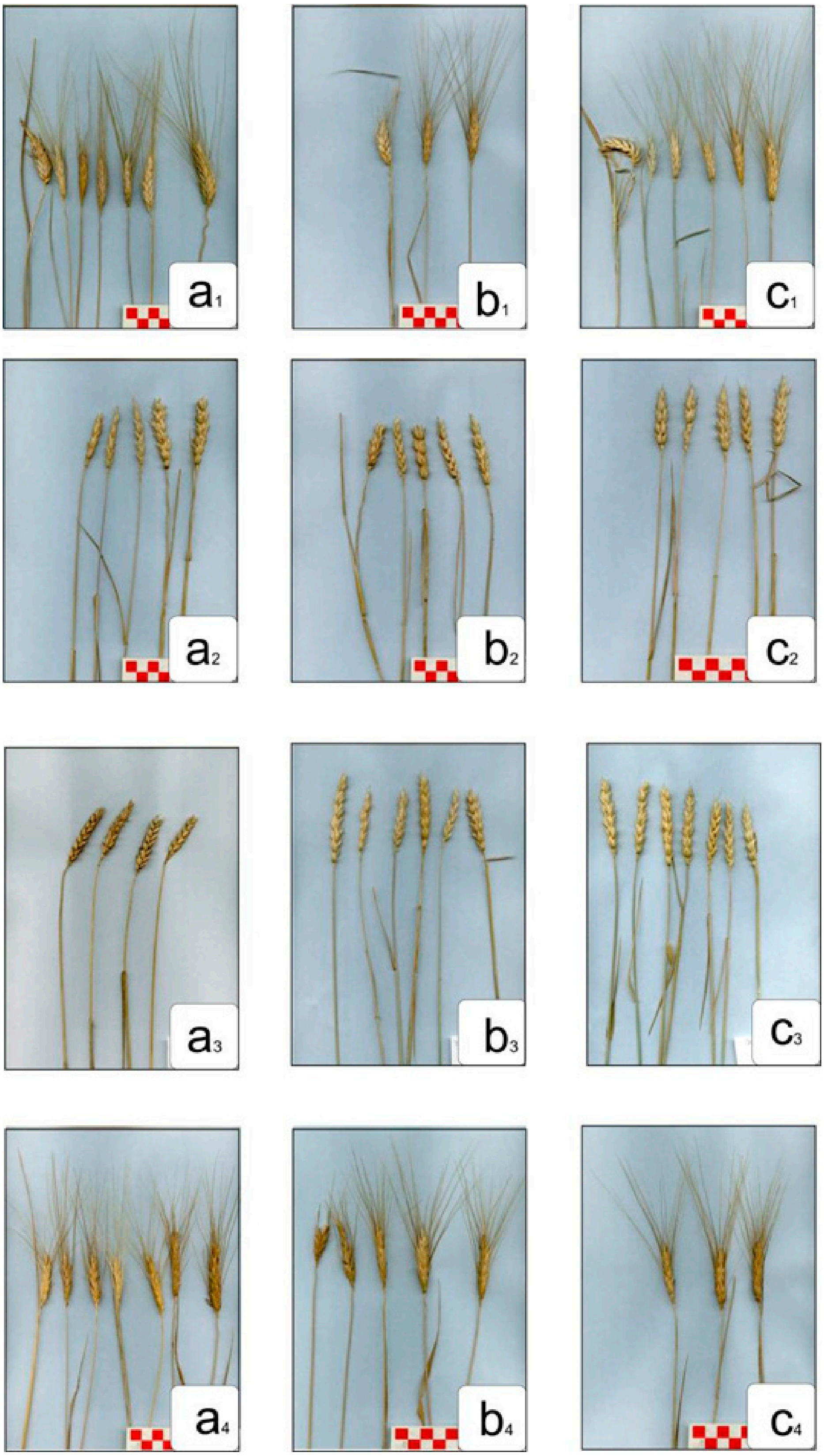

a – control; b – 1.10 g L^{-1} NaCl; c – 0.40 g L^{-1} Na_2SO_4

First upper row (1) – cv Zolotaya; second row (2) – cv Orenburgskaya 10; third row (3) – cv Orenburgskaya 23; bottom row (4) – cv Ulyanovskaya 105.

Table S1. The number of kernels according to the variants of experience.

Wheat cultivars	Salinity variants	Location in the spikelet	Experiment variant number	Number of kernels after parsing all spikes	Number of fully and partially matured kernels	Number of fully matured kernels to test for asymmetry
Zolotaya	Control	Left	1	49	36	15
		Middle	2	14	9	6
		Right	3	51	31	15
	NaCl	Left	4	18	17	15
		Middle	5	7	7	7
		Right	6	18	16	15
	Na_2SO_4	Left	7	37	34	15
		Middle	8	14	13	10
		Right	9	43	38	15
	In total					113
Orenburgskaya 10	Control	Left	10	25	18	15
		Middle	11	5	5	5
		Right	12	19	11	11
	NaCl	Left	13	23	13	11
		Middle	14	10	9	9
		Right	15	24	17	14
	Na_2SO_4	Left	16	16	11	11
		Middle	17	4	3	3
		Right	18	21	18	14
	In total					93
Ulyanovskay	Control	Left	19	66	48	15

a 105		Middle	20	35	29	10
		Right	21	68	44	15
	NaCl	Left	22	60	60	15
		Middle	23	17	17	12
		Right	24	56	53	15
	Na_2SO_4	Left	25	68	60	15
		Middle	26	26	25	15
		Right	27	70	65	15
	In total					127
Orenburgskaya 23	Control	Left	28	45	38	15
		Middle	29	26	23	15
		Right	30	44	34	14
	NaCl	Left	31	43	40	15
		Middle	32	21	19	14
		Right	33	40	37	15
	Na_2SO_4	Left	34	40	30	15
		Middle	35	8	8	7
		Right	36	41	34	15
		In total				125
